# Use of a High-Density Protein Microarray to Identify Autoantibodies in Subjects with Type 2 Diabetes Mellitus and an HLA Background Associated with Reduced Insulin Secretion

**DOI:** 10.1371/journal.pone.0143551

**Published:** 2015-11-25

**Authors:** Douglas C. Chang, Paolo Piaggi, Robert L. Hanson, William C. Knowler, John Bucci, Guene Thio, Maximilian G. Hohenadel, Clifton Bogardus, Jonathan Krakoff

**Affiliations:** 1 Phoenix Epidemiology and Clinical Research Branch, National Institute of Diabetes and Digestive and Kidney Diseases, National Institutes of Health, Phoenix, Arizona, United States of America; 2 Life Technologies, Carlsbad, California, United States of America; Duke University Medical Center, UNITED STATES

## Abstract

New biomarkers for type 2 diabetes mellitus (T2DM) may aid diagnosis, drug development or clinical treatment. Evidence is increasing for the adaptive immune system’s role in T2DM and suggests the presence of unidentified autoantibodies. While high-density protein microarrays have emerged as a useful technology to identify possible novel autoantigens in autoimmune diseases, its application in T2DM has lagged. In Pima Indians, the HLA haplotype (HLA-DRB1*02) is protective against T2DM and, when studied when they have normal glucose tolerance, subjects with this HLA haplotype have higher insulin secretion compared to those without the protective haplotype. Possible autoantibody biomarkers were identified using microarrays containing 9480 proteins in plasma from Pima Indians with T2DM without the protective haplotype (n = 7) compared with those with normal glucose regulation (NGR) with the protective haplotype (n = 11). A subsequent validation phase involving 45 cases and 45 controls, matched by age, sex and specimen storage time, evaluated 77 proteins. Eleven autoantigens had higher antibody signals among T2DM subjects with the lower insulin-secretion HLA background compared with NGR subjects with the higher insulin-secretion HLA background (p<0.05, adjusted for multiple comparisons). PPARG2 and UBE2M had lowest p-values (adjusted p = 0.023) while PPARG2 and RGS17 had highest case-to-control antibody signal ratios (1.7). A multi-protein classifier involving the 11 autoantigens had sensitivity, specificity, and area under the receiver operating characteristics curve of 0.73, 0.80, and 0.83 (95% CI 0.74–0.91, p = 3.4x10^-8^), respectively. This study identified 11 novel autoantigens which were associated with T2DM and an HLA background associated with reduced insulin secretion. While further studies are needed to distinguish whether these antibodies are associated with insulin secretion via the HLA background, T2DM more broadly, or a combination of the two, this study may aid the search for autoantibody biomarkers by narrowing the list of protein targets.

## Introduction

In contrast to Type 2 diabetes mellitus (T2DM), Type 1 diabetes mellitus (T1DM) is well-recognized as an autoimmune disease resulting from immune-mediated pancreatic beta-cell destruction and is associated with clinically useful autoantigen biomarkers [1, 2]. T2DM is traditionally regarded as a metabolic disease, with a defect in insulin action preceding or concurrent with pancreatic beta-cell failure [3]. However, the immune system is increasingly recognized as a potential pathogenic component of T2DM and its most important risk factor, obesity [4–6]. Serum concentrations of gamma globulin, a nonspecific measure of the humoral immune system, were positively associated with development of T2DM in Pima Indians [7]. Diminished obesity-associated insulin action is characterized by chronic inflammation involving infiltration of macrophages and both T and B cells into adipose tissue [8]. In a mouse model, B cells appeared to play an instrumental role in worsening insulin action via modulation of T cells and production of pathogenic IgG antibodies, indicating a role for adaptive immunity in the pathophysiology of T2DM [4]. In addition, a subgroup of patients with phenotypic T2DM has measurable antibody titers and islet cell reactive T-cells, both attributes of adaptive immune response [9–13]. However not all such patients with an islet reactive T cell response had previously-described T1DM-associated autoantibodies [10]. Autoantibodies have been detected in subgroups of patients with T2DM who were at increased risk for hypertension or cardiovascular complications (G-protein coupled receptors [14]) and who had maculopathy and macroalbuminuria (rho-kinases [15]). In addition, IL-6 autoantibodies have been detected in sera from 2.5% of Danish patients [16].

Many autoimmune diseases show an association with certain HLA haplotypes, usually involving the major histocompatibility complex class II which encodes for genes that are important for immune response regulation [17]. An HLA haplotype (HLA-DRB1*02) protective for T2DM and associated with increased insulin secretion was identified among Pima Indians, a group with high rates of T2DM and obesity but low prevalence of GAD2 and other known islet cell antibodies. These findings were interpreted as supporting a potential role for loss of self-tolerance [18, 19].

Functional protein microarrays, which have large numbers of correctly folded and functional proteins, have emerged as a useful biotechnology to identify novel autoantigens in a number of different diseases including T1DM [2], cancer [20], neuromyelitis optica [21], and several rheumatologic conditions [22, 23].

The aim of this study was to identify potential novel autoantibodies for T2DM using a functional protein microarray containing 9480 human proteins with plasma from Pima Indians with type 2 diabetes and the lower insulin-secretion HLA background (18) (i.e. without HLA-DRB1*02) compared with those with normal glucose regulation and the higher insulin-secretion haplotype (i.e. with HLA-DRB1*02). This was followed by a validation study with a selected subset of antigens and included antigens identified as being associated with T1DM [1, 24] in a second larger cohort.

## Materials and Methods

### Subjects

**Fig 1:**
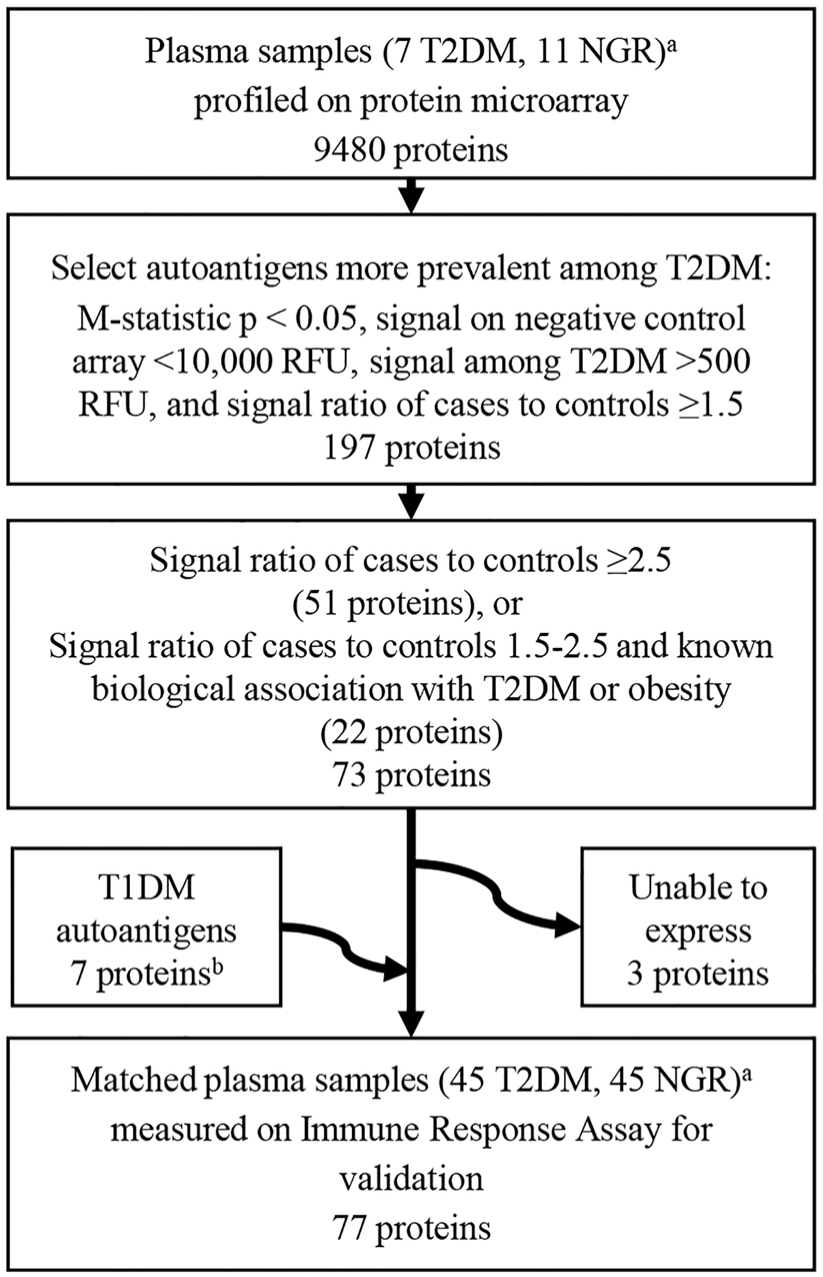
Selection of proteins associated with type 2 diabetes mellitus. T2DM = type 2 diabetes mellitus; NGR = normal glucose regulation; T1DM = type 1 diabetes mellitus; ^a^ T2DM volunteers were without the higher insulin-secretion HLA-DRB1*02 haplotype and NGR volunteers had the higher insulin-secretion haplotype. ^b^ IAPP, HSP60, SLC30A8, PTPRN, insulin, CPE, GAD2.

Our study involved two separate cohorts from studies approved by the Institutional Review Board of the National Institute of Diabetes and Digestive and Kidney Diseases (NIDDK). All subjects provided written informed consent before participation. For the first cohort, volunteers participated in a previously described longitudinal inpatient study, initiated in 1982, examining risk factors for T2DM at the Obesity and Diabetes Clinical Research Section, NIDDK, Phoenix AZ [25]. All subjects were between 18 and 50 years of age, nonsmokers, without prior diagnosis of diabetes and other serious medical problems (e.g. autoimmune, heart, cerebrovascular diseases) at the baseline admission or at subsequent admissions (subjects invited back for re-admission annually), and were not taking any medications. Upon admission, volunteers were fed a weight maintaining diet. On day 4, a 75 gram oral glucose tolerance test (OGTT) was administered. Glucose concentrations were measured using glucose oxidase method (Beckman Instruments, Fullerton CA). Plasma specimens from 18 Pima Indian volunteers, 7 from full-heritage Pima males with T2DM with the lower insulin-secretion HLA background (i.e. without HLA-DRB1*02 haplotype) and 11 from individuals with normal glucose regulation with the higher insulin-secretion HLA haplotype, were profiled on protein microarrays containing 9480 human proteins (Fig 1). For the first cohort used in this microarray study, T2DM specimens were restricted to the OGTT which initially diagnosed T2DM and glucose regulation status was determined by an OGTT according to the 2003 criteria of the American Diabetes Association (ADA) [26].

For the second cohort, subjects participated in a previously described longitudinal study of the etiology of T2DM among the Gila River Indian Community in Arizona, where most of the residents are Pima Indians or Tohono O’odham, a closely related tribe [27]. Subjects aged five years and over were examined approximately every two years, which included a 75 gram OGTT. For the present study, eligible specimens were associated with subjects aged 18 years or greater at the time of specimen collection. Plasma specimens from 45 case patients with T2DM with the lower insulin-secretion HLA background (i.e. without the HLA-DRB1*02 haplotype) were pair-wise matched by age, sex, and storage time (since these variables may influence antibody levels) with 45 control patients with normal glucose regulation with the higher insulin-secretion HLA haplotype. Similar to the first cohort, plasma specimens from T2DM subjects in the second cohort were restricted to those from the initial OGTT which diagnosed T2DM and glucose regulation status was determined by an OGTT according to the 2003 ADA criteria [26].

### Protein Selection

Of the 9480 proteins initially profiled in the first phase of the study, 197 proteins were identified which met these statistical criteria: 1) M-statistic p-value <0.05, 2) signal on the negative control array <10,000 RFU, 3) average signal of cases >500 RFU, and 4) signal ratio of cases to controls ≥1.5 (Fig 1). We then chose the top proteins based on signal ratio of cases to controls greater than ≥2.5 and those with signal ratio 1.5–2.5 in which biological relevance to diabetes mellitus or obesity was inferred based on literature review (JK). Of 73 proteins meeting the above criteria, three proteins (ABI1, ACVR1C, and MAP2K3 transcript variant A) did not meet quality control standards for protein expression and were excluded. We also included 7 autoantigens associated with T1DM: (a) islet amyloid polypeptide (IAPP), (b) heat shock protein 60 (HSP60), (c) solute carrier family 30 (zinc transporter), member 8 (SLC30A8, alias ZnT8)), (d) protein tyrosine phosphatase, receptor type (PTPRN, alias IA-2), (e) insulin, (f) caboxypeptidase E (CPE), (g) glutamate decarboxylase 2 (GAD2) [24]. Insulin, HSP60, IAPP and GAD2 proteins were also among the initial 9480 proteins. In the end, seventy-seven proteins were selected for confirmatory testing.

### Initial Experiment with 9480 Proteins (First Cohort)

Human clones were obtained from Invitrogen’s Ultimate open reading frame (ORF) collection or from a Gateway collection of kinase clones developed by Protometrix. The nucleotide sequence of each clone was verified by full length sequencing. All clones were transferred into a system for expressing recombinant proteins in insect cells via baculovirus infection. ProtoArray Human Protein Microarray v5.0, contains 9480 glutathione S-transferase-tagged proteins printed on a nitrocellulose-coated slide arrayed in 48 subarrays. Proteins were non-covalently attached on slides which allows for random orientation in native conformations. Protein microarrays were incubated with 18 human plasma samples previously stored at -20°C at a 1:500 dilution. Reactions were detected by Alexa-Fluor-647-conjugated anti-human IgG. Images were scanned with GenePix 6.0 software.

### Confirmatory Experiment (Second Cohort)

The Luminex system, similar to ELISA, was used to test plasma previously stored at -70°C from the 45 cases and 45 controls in the second cohort. Of the 77 proteins, 72 proteins were captured on anti-GST conjugated MagPlex beads and 5 proteins were directly conjugated to unique bead regions, each bead with a unique spectral signature. The beads were incubated with experimental samples at a 1:200 dilution of plasma for one hour followed by incubation with biotinylated anti-human IgG for one hour. After removal of excess biotinylated anti-human IgG, streptavidin conjugated to fluorescent protein R-Phycoerythrin (Streptavidin-RPE) was added and incubated for 30 minutes. After washing to remove unbound Streptavidin-RPE), the beads were analyzed with Luminex 200 to measure signal strength for each protein, tested in triplicate.

### Statistical Analysis

In the first phase of the study, data were normalized using the Robust Linear Model [28]. M-statistics analysis, which compares order of values between 2 groups and determines the chance of the order occurring randomly, was applied [29]. M-statistics were used to calculate p-values and to establish signal cutoffs for determining antibody positivity using the following analysis parameters: signals above background threshold of 500MFI and signal difference between two signals>200MFI. Using a non-informative prior distribution of prevalence and acknowledging a binomial sampling scheme (Bayesian estimator) [29], the prevalence of an autoantigen was calculated p^=(count+1)/(n+2).

In the second cohort, signals were log10-transformed to approximate a normal distribution. Mixed models were used to compare cases and controls, accounting for the matched design as fixed effect and triplicate measures (see above) as random effect. In these mixed models carried out on the 77 protein signals of the validation phase, p-values were one-sided to confirm the directionality of relationships obtained in the previous first cohort since we had deliberately chosen proteins with signals more elevated among cases than controls. To account for multiple testing (i.e. 77 proteins), p-values were adjusted according to the Benjamini & Hochberg method [30] using a false discovery rate (FDR) of 0.05. Both mixed models and FDR analysis were performed in SPSS v22. Similar to the first cohort, M-statistics were applied in a similar fashion to establish cut-offs and case counts as well as estimate prevalence.

The top eleven statistically significant protein targets based on mixed models in the second cohort were then added to a recursive feature selection algorithm to assess the predictive performance of distinguishing disease from healthy samples of several classification models. The feature selection algorithm was written in the R language (http://www.R-project.org/) and relying on techniques implemented by the classification and regression training package (caret: http://caret.r-forge.r-project.org/). A naive Bayes classification technique was used throughout the feature selection algorithm which relies on Bayesian probability and assumes independence between predictors (i.e. protein signals). Log-transformed signals were used. Three protein signals were correlated with each other across samples and were highly correlated (Pearson r > 0.70) with other proteins. The classification was run both with and without these correlated proteins. These correlated proteins did not have a major impact on performance and were left in the feature selection process. The naive Bayes method also assumes a Gaussian distribution when making class predictions. The feature selection algorithm used a backwards selection process with a 10-fold cross-validation sampling. The algorithm was run on all 11 protein targets, as well as random subsets of 5, 4 and 3 proteins and repeated five times with the goal to find the subset of protein targets that can be used to distinguish healthy from disease samples with highest accuracy. For each iteration, a naive Bayes classifier was first fitted to all the predictors (protein biomarkers). The predictors were then ranked by their individual contribution to the classification performance by the area under the receiver operating characteristic curve. In subsequent iterations, less important predictors dropped one at a time prior to re-fitting the model. We chose to maximize the sum of the sensitivity metric (percentage of true positives) plus the specificity (percentage of true negatives) to assess overall classifier performance and optimal model selection.

## Results

Of 9480 proteins screened, 197 proteins met all of initial selection criteria: p-value <0.05, negative control signal<10,000 RFU, signal >500 RFU, and signal ratio of cases to controls greater than 1.5 (Fig 1). Of these 197 proteins, 51 proteins had signal ratio of cases to controls ≥2.5. Of those with average signal ratio 1.5–2.5 (146 proteins), we selected 22 additional proteins based on biological importance to obesity or diabetes mellitus. In the second cohort, T2DM subjects had significantly higher BMI than those with normal glucose regulation (41kg/m^2^ vs. 33 kg/m^2^, p<0.001) (Table 1).

**Table 1:** Subject characteristics.

	First cohort (unmatched)			Second cohort (matched)^a^		
Characteristic	T2DM^b^ (n = 7)	NGR^c^ (n = 11)	p-value^d^	T2DM^b^ (n = 45)	NGR^c^ (n = 45)	p-value^e^
Age, years (range)	34±8 (23–46)	31±8 (21–45)	0.50	34 ± 11 (18–64)	34 ± 11 (18–63)	0.06
Male sex (%)	7 (100)	11 (100)	-	23 (51)	23 (51)	-
Plasma storage, years (range)	18.2 ± 2.3 (15.4–21.0)	17.6 ± 2.4 (13.0–21.2)	0.60	16.9 ±3.8 (7.7–22.1)	16.7 ± 4.0 (8.2–22.4)	0.32
BMI, kg/m^2^ (range)	37 ± 5 (30–42)	36 ± 8 (20–51)	0.74	41 ± 8 (27–60)	33 ± 7 (20–49)	<0.001
Height, cm (range)	170 ± 4 (166–176)	171 ± 6 (158–179)	0.92	167 ± 8 (153–191)	166 ± 7 (151–181)	0.07
Weight, kg (range)	108 ± 17 (82–125)	105 ± 26 (50–147)	0.81	114 ± 21 (78–165)	91 ± 23 (55–148)	<0.001
Fasting plasma glucose, mg/dl (range)	118 ± 20 (97–157)	85 ± 10 (60–96)	<0.001	183 ± 69 (97–309)	90 ± 5.5 (79–99)	<0.0001
2-Hour glucose, mg/dl (range)	261 ± 28 (225–317)	106 ± 14 (88–130)	<0.0001	319 ± 96 (201–552)	102 ± 18 (61–139)	<0.0001

T2DM, type 2 diabetes mellitus; NGR, normal glucose regulation.

^a^ Matched by age, sex and plasma storage time.

^b^ Without higher insulin-secretion HLA-DRB1*02 haplotype.

^c^ With higher insulin-secretion HLA-DRB1*02 haplotype.

^d^ Unpaired t-test.

^e^ Paired t-test.

**Fig 2:**
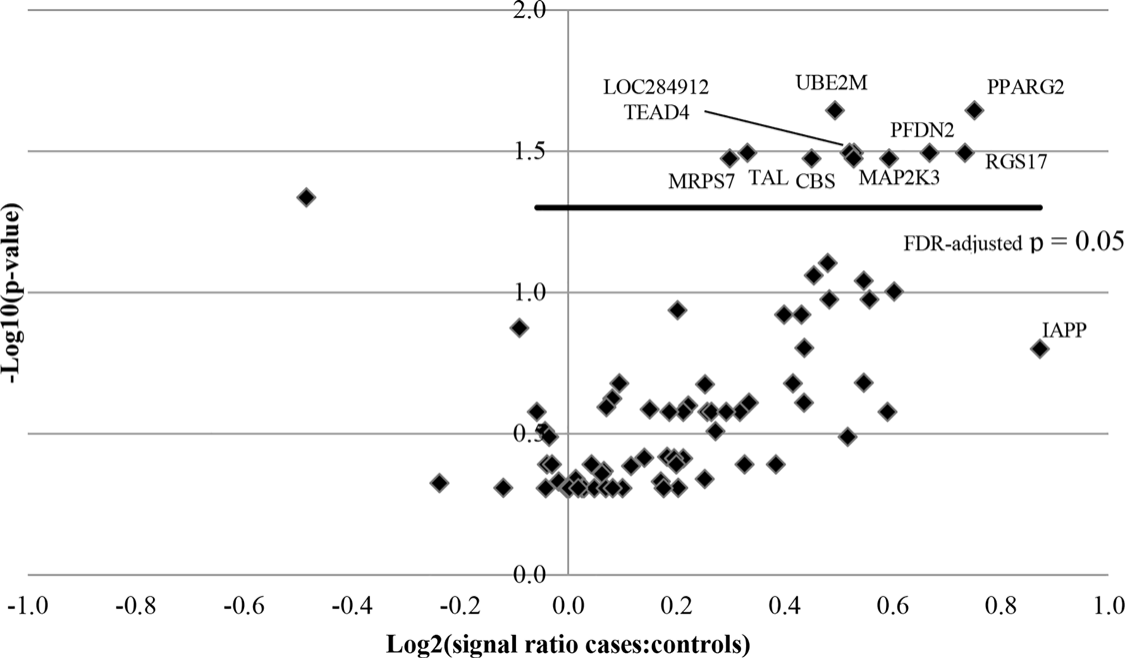
Volcano plot of 77 proteins. FDR = False Discovery Rate. Lower p-values are towards the top. The statistically significant p-values are above the line. The proteins which have higher case-to-control signal ratios are further towards the right.

In the second cohort (Table 2 and S1 Table), 11 of the 77 proteins (14%) were elevated in cases vs. controls (p< 0.05 adjusted for multiple comparisons using FDR of 0.05, Table 2). The estimated prevalence of PPARG2 autoantibodies was 17% among cases and 2% among controls (Table 2). Estimated autoantibody prevalence among cases was highest for TAL and MRPS7 (79% and 40%, respectively; Table 2). PPARG2 and UBE2M had the lowest adjusted p-values (p = 0.023, Table 2, Fig 2). Of the 77 proteins, PPARG2 and RGS17 had the second to the highest case-to-control signal ratios (1.7) (Table 2, Fig 2).

**Table 2:** Eleven statistically significant proteins with false discovery rate adjusted p-value<0.05 and case to control signal ratio >1.0 in validation cohort.

Protein	Database ID	1^st^ Cohort		2^nd^ Cohort—Validation					
		p-value	Signal ratio	Beta	SE	FDR adjusted p-value	Signal ratio	Case prev	Control prev
PPARG2	NM_015869.2	0.047	1.8	-0.147	0.044	0.023	1.7	17%	2%
UBE2M	BC058924.1	0.043	2.9	-0.118	0.036	0.023	1.4	26%	6%
TAL	BC018847.1	0.001	1.8	-0.118	0.039	0.032	1.3	79%	55%
LOC284912	BC001801.1	0.011	3.0	-0.092	0.032	0.032	1.4	13%	2%
TEAD4	NM_201443.1	0.043	2.9	-0.109	0.038	0.032	1.4	13%	2%
RGS17	NM_012419.3	0.043	2.6	-0.143	0.051	0.032	1.7	34%	15%
PFDN2	NM_012394.2	0.039	4.3	-0.112	0.041	0.032	1.6	19%	6%
MRPS7	NM_015971.2	0.047	3.4	-0.095	0.036	0.034	1.2	40%	26%
OSBPL11	NM_022776.3	0.047	2.0	-0.103	0.039	0.034	1.4	15%	2%
CBS	NM_000071.1	0.039	1.7	-0.101	0.039	0.034	1.4	9%	2%
MAP2K3-B	NM_145109.1	0.043	1.8	-0.125	0.048	0.034	1.5	19%	6%

Beta coefficient for group comparisons expressed as a multiplier (i.e. log value). SE, standard error; FDR, false discovery rate; prev, prevalence.

**Fig 3:**
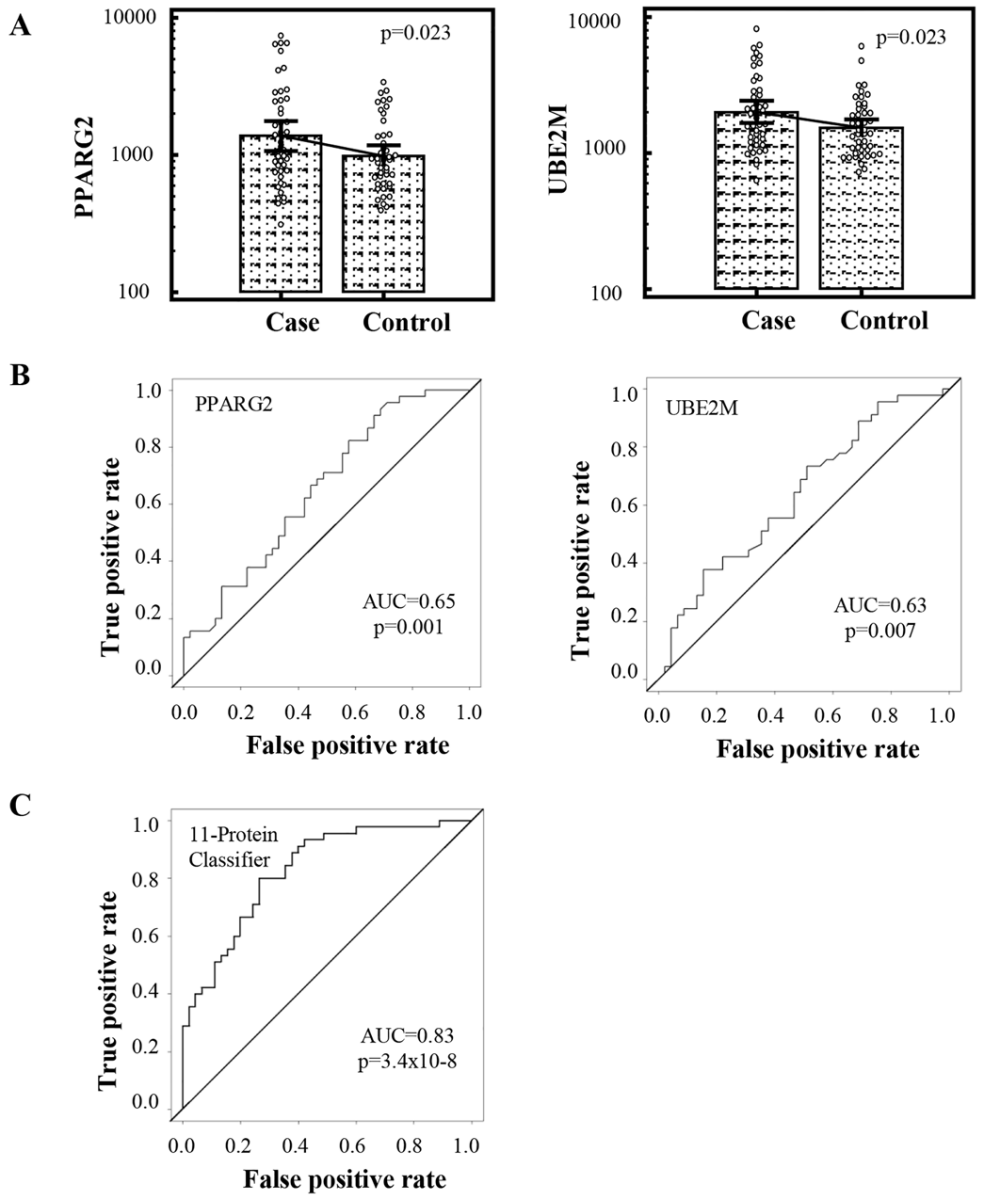
PPARG2, UBE2M, and 11-protein classifier. (A) Relative signals of PPARG2 and UBE2M. (B) Receiver operating characteristic (ROC) analysis representing the accuracy (AUC) of PPARG2 and UBE2M. (C) ROC analysis of 11 statistically significant proteins.

Signals for PPARG2 and UBE2M for T2DM and NGR subjects in the second cohort are shown in Fig 3A. The area under the receiver operating characteristic curve (AUC) for PPARG2 and UBE2M were 0.65 (p = 0.001) and 0.63 (p = 0.007), respectively (Fig 3B).

Individually, PPARG2 had sensitivity 0.42, specificity 0.78, accuracy 0.60, and AUC 0.65 (95% CI 0.54–0.76) (Table 3), UBE2M had similar specificity (0.82), accuracy (0.58) and AUC (0.63, 95% CI 0.52–0.75) but lower sensitivity (0.33) (Table 3). A combination classifier involving both PPARG2 and UBE2M had a higher sensitivity (0.51), specificity (0.82), accuracy (0.67) and AUC (0.70, 95% CI 0.59–0.81) than either of them individually (Table 3). A model involving all 11 statistically significant proteins (Table 2) had sensitivity 0.73, specificity 0.80, accuracy 0.77, AUC 0.83 (p = 3.4x10^-8^, 95% CI 0.74–0.91) (Fig 3C and Table 3).

**Table 3:** Sensitivity and specificity of PPARG2, UBE2M, and 11-protein classifier.

Protein	Sensitivity	Specificity	Accuracy	AUC (95% CI)	p-value
PPARG2	0.42	0.78	0.60	0.65 (0.54–0.76)	0.001
UBE2M	0.33	0.82	0.58	0.63 (0.52–0.75)	0.007
PPARG2 and UBE2M	0.51	0.82	0.67	0.70 (0.59–0.81)	0.0002
11 protein classifier	0.73	0.80	0.77	0.83 (0.74–0.91)	3.4x10-8

AUC, area under the curve; CI, confidence interval.

In mixed models further adjusting for BMI which evaluated the 11 statistically significant proteins, adjusted p-values for UBE2M, LOC284912, PFDN2 and OSBPL11were 0.019, 0.021, 0.021, and 0.049, respectively, whereas the remaining seven proteins (PPARG2, TAL, TEAD4, RGS17, MRPS7, CBS, and MAP2K3) were not statistically significant (p>0.05).

Signals for T1DM associated autoantigens (IAPP, HSP60, SLC30A8, PTPRN, CPE, GAD2, insulin) were not significantly elevated among cases compared with matched controls (p>0.05). IAPP case-to-control signal ratio was greatest among all 77 proteins though the difference was not statistically significant (ratio 1.8, p = 0.16; Fig 2). IAPP was also among the initial 9480 proteins tested in the first cohort but did not have elevated case to control signal ratio (1.0) and was not statistically significant (p = 0.64).

## Discussion

Using a protein microarray containing 9480 human proteins, we found 197 autoantibodies to proteins which were significantly elevated in Pima Indian men with T2DM and lower insulin-secretion HLA background compared with those with normal glucose regulation and the higher insulin-secretion HLA background. We then selected 77 proteins for confirmation in a second cohort matched for age, sex and storage time. Eleven autoantibodies were significantly elevated in cases versus controls in the second cohort, including proteins in which genetic polymorphisms had been associated with diabetes or obesity in Pima Indians (anti-PPARG2 and anti-MAP2K3, respectively).

We identified the presence of PPARG2 antibodies among case patients with T2DM and the lower insulin-secretion HLA background. It has been proposed that progression from innate to adaptive immune response in obesity and T2DM occurs through release of cryptic antigens through adipocyte cell death induced by innate inflammation in response to fatty acids [8]. Since PPARG2 is highly localized to adipose tissue [31], PPARG2 is a promising candidate for such an autoantigen. Expression of PPARG may be directly involved in the leptin-induced adipocyte apoptosis signaling [32, 33]. In the Pima Indian population, a Pro12Ala functional variant is associated with several metabolic predictors of T2DM including whole-body and hepatic insulin action and mean fasting plasma insulin [34]. PPARG is a nuclear receptor which serves as a key regulator of adipogenesis. Genetic mutations or polymorphisms and altered signaling or expression of PPARG has been associated with decreased insulin action, obesity, dyslipidemia, as well as cardiovascular disease and cancer [31]. The thiazolidinediones, a drug class used to treat T2DM by improving insulin action on liver, adipose and muscle, are synthetic ligands which activate PPARs with highest specificity for PPARG.

Antibodies to MAP2K3-B were also significantly associated with cases in our study. Adipogenesis is regulated by MAP kinase signaling cascades such as the p38 MAP kinase pathway. MAP2K3 functions as a specific upstream activator of p38 isoforms [35]. Moreover, overexpression of MAP2K3 leads to modest increase in KLF9 which serves as a key pro-adipogenic transcription factor through regulation of PPARG2 expression [35, 36]. Polymorphisms in MAP2K3 have previously been found to be associated with BMI in both Caucasians and American Indians [35]. Since MAP2K3 expression levels in adipose tissue were also positively correlated with BMI [35], MAP2K3-B release after adipocyte death could also lead to loss of self-tolerance to this autoantigen, increased adaptive immune response (abnormal B cell activation), and subsequent decreased insulin action [4, 8]. Moreover, associations with MAP2K3 and PPARG2 antibodies were attenuated after adjustment for BMI, supporting a hypothesis that obesity itself (with or without diabetes) may produce an adaptive immune response.

In addition to MAP2K3-B and PPARG2, autoantibodies to OSBPL11, CBS and TAL were also associated with cases and may be relevant to the pathophysiology of T2DM or obesity. OSBPL11 belongs to a group of intracellular lipid receptors and polymorphisms have been associated with T2DM in humans and components of the metabolic syndrome [37]. Hepatic CBS, an enzyme involved in homocysteine metabolism, has been found to be elevated in a diabetic rat model [38]. TAL is involved in the pentose phosphate pathway of carbohydrate metabolism which provides an alternative pathway for glucose oxidation [39].

We also identified antibodies to UBE2M. Through modification of proteins with ubiquitin, UBE2M plays an essential role within the ubiquitin-proteasome system which is responsible for targeting abnormal or short-lived proteins for degradation and may also have a role in T2DM [40, 41]. For example, defective protein degradation in beta cells of T2DM, mediated in part by abnormally folded human IAPP may compromise beta cell viability [42]. There is also evidence for the role of proteasome dysregulation in microvascular complications of T2DM [43].

To our knowledge, PFDN2 has not been previously associated with T2DM. PFDN2 is one of the subunits of prefoldin, a molecular chaperone which facilitates proper protein folding through binding to newly unfolded proteins or through preventing protein aggregation. There is evidence that prefoldin prevents formation of pathogenic Huntington protein aggregates [44]. Moreover, knockdown of PFDN2 caused accumulation of ubiquitinated aggregated α-synuclein, a protein involved mechanistically in the pathogenesis of Parkinson’s disease through protein misfolding, formation of abnormal oligomers, amyloid fibrils and Lewy bodies [45]. Similar to Huntington’s and Parkinson’s diseases, T2DM is a disease involving protein aggregation with amyloid features. In T2DM, IAPP oligomers are suspected to be toxic to beta-cells and lead to formation of fibrils and pancreatic amyloid [46]. The identification of PFDN2 autoantibodies in our patients and the known biology of prefoldin from other diseases may indicate a role for prefoldin in T2DM which could be explored through functional studies. Antibodies to other proteins, including LOC284912, TEAD4, RGS17, and MRPS7, were also identified by our study; to our knowledge, the roles of these proteins in T2DM are uncertain and may present opportunities for exploration.

Two different assays were used in our study, supporting that these results are not an assay specific artifact. Moreover, the recombinant proteins used in our assays were expressed in insect cells via baculovirus infection which have several characteristics that are usually advantageous, including being frequently soluble, post-translationally modified, correctly folded, and active [29]. Recently, antibodies to IAPP oligomers were detected in a small number of patients with T2DM [47]. Although the signal ratio for IAPP was high at 1.8, the difference was not significant. Due to the type of assay used, the presence of oligomer antibodies was not evaluated.

Although M-statistics may not be universally agreed upon as an estimator of prevalence, our main findings were confirmed through mixed models which accounted for the paired design and samples run in triplicate. The use of M-statistics has been used successfully to identify autoantigens in other diseases including T1DM [2]. M-statistics allowed us to define a cutoff for the presence or absence of autoantibody in a particular patient sample and estimate the prevalence of the autoantibody, an important estimate for planning further studies [29]. Although prevalence estimates for several significant proteins in this study were lower than other T1DM autoantibodies, such as GAD2 with a prevalence of about 80% in new-onset T1DM [2, 48], clinically important indicators of disease progression need not have high prevalence. For example, receptor tyrosine kinase Her2 is found in approximately 25% of all breast cancers and is clinically important as an indicator of disease progression and guides therapeutic options [49].

Our study has limitations. The major limitation is that in both cohorts, participants differed by both diabetes status and the HLA-DRB1*02 haplotype associated with higher insulin secretion [18]. This means that we cannot distinguish whether the antibody associations are due to T2DM more broadly, the decreased insulin-secretion HLA background, or a combination of the two. However, it is important to note that the HLA background was found to be associated with decreased insulin secretion within the Pima Indian population [18]. Impaired insulin secretion is critical in the pathogenesis of T2DM and reduced insulin secretion is a risk factor for T2DM even when subjects have normal glucose regulation [3, 50]. Thus if these autoantibodies are associated with the HLA background itself rather than T2DM, then it may indicate a role for these autoantibodies in decreased beta cell function via a reduction in insulin secretion and may suggest a role for these autoantibodies in a pre-diabetic stage. In the next phase, we will investigate whether these 11 statistically significant autoantibodies are associated with T2DM independent of the HLA background. The other major limitation is that this study was performed in a population with a high risk of diabetes, and although the pathophysiology of the development of diabetes in Pima Indians mirrors those of other populations, the autoantibodies described may be unique to this population.

In summary, by initial screening of 9480 proteins followed by a confirmatory study, we identified the presence of 11 novel autoantibodies which were associated with phenotypic T2DM and a specific HLA genotype associated with insulin secretion. While further studies are needed to confirm and distinguish whether these antibodies are associated with decreased insulin secretion via the HLA background, T2DM more broadly, or a combination of both, this study may aid the search for autoantibody biomarkers by identifying a potential list of protein targets.

## Supporting Information

S1 Table Sixty-six proteins with false discover rate adjusted p-value>0.05 or case to control ratio≤1.0 in validation cohort. Proteins in bold refer to type 1 diabetes mellitus associated autoantigens. FDR, false discovery rate.
(DOCX)
