# Supplementary material for: Use of a High-Density Protein Microarray to Identify Autoantibodies in Subjects with Type 2 Diabetes Mellitus and an HLA Background Associated with Reduced Insulin Secretion
Source: PLoS One. 2015 Nov 25;10(11):e0143551. doi: 10.1371/journal.pone.0143551 (PMC4659622; doi:10.1371/journal.pone.0143551)
Supplement: S1 Table — Proteins in bold refer to type 1 diabetes mellitus associated autoantigens. FDR, false discovery rate. (DOCX) [file pone.0143551.s001.docx]

**S1 Table**. Sixty-six proteins with false discover rate adjusted p-value>0.05 or case to control ratio≤1.0 in validation cohort. Proteins in bold refer to type 1 diabetes mellitus associated autoantigens. FDR, false discovery rate.

|  |  | 1^st^ Cohort | | 2^nd^ Cohort - Validation | |
| --- | --- | --- | --- | --- | --- |
| Protein | Database ID | p-value | Signal ratio | FDR adjusted  p-value | Signal  ratio |
| **heat shock protein 60** | **HSP60** | - | - | 0.046 | 0.7 |
| D-tyrosyl-tRNA deacylase 1 homolog (S. cerevisiae) (DTD1) | BC045167.2 | 0.043 | 2.7 | 0.079 | 1.4 |
| MHC class I polypeptide-related sequence A (MICA) | BC016929.1 | 0.039 | 1.8 | 0.087 | 1.4 |
| Oxysterols receptor LXR-alpha | BC041172.1 | 0.043 | 2.1 | 0.091 | 1.5 |
| interleukin 1, beta (IL1B) | NM_000576.1 | 0.043 | 1.8 | 0.099 | 1.5 |
| Rho guanine nucleotide exchange factor (GEF) 1 (ARHGEF1), transcript variant 2 | NM_004706.3 | 0.011 | 2.7 | 0.106 | 1.4 |
| cystatin B (stefin B) (CSTB) | NM_000100.2 | 0.025 | 1.9 | 0.106 | 1.5 |
| septin 11 (SEPT11) | NM_018243.1 | 0.047 | 3.0 | 0.116 | 1.2 |
| 3-hydroxybutyrate dehydrogenase, type 2 (BDH2) | NM_020139.1 | 0.039 | 2.5 | 0.120 | 1.3 |
| NCK adaptor protein 1 (NCK1) | NM_006153.3 | 0.043 | 1.9 | 0.120 | 1.3 |
| protein regulator of cytokinesis 1 (PRC1), transcript variant 1 | NM_003981.2 | 0.010 | 3.0 | 0.134 | 0.9 |
| **Homo sapiens, carboxypeptidase E (CPE), mRNA** | **NM_001873.1** | - | - | 0.158 | 1.4 |
| **islet amyloid polypeptide (IAPP)** | **NM_000415.1** | 0.641 | 1.0 | 0.159 | 1.8 |
| Protein DGCR6L | BC000682.1 | 0.043 | 5.2 | 0.209 | 1.5 |
| cytoskeleton associated protein 2 (CKAP2) | BC010901.1 | 0.011 | 7.4 | 0.210 | 1.3 |
| Isovaleryl-CoA dehydrogenase, mitochondrial | BC017202.2 | 0.011 | 3.2 | 0.210 | 1.1 |
| mediator of RNA polymerase II transcription, subunit 8 homolog (S. cerevisiae) (MED8), transcript variant 4 | NM_001001653.1 | 0.009 | 2.9 | 0.212 | 1.2 |
| Neuronal migration protein doublecortin | NM_178152.1 | 0.011 | 1.6 | 0.238 | 1.1 |
| proopiomelanocortin (adrenocorticotropin/ beta-lipotropin/ alpha-melanocyte stimulating hormone/ beta-melanocyte stimulating hormone/ beta-endorphin) (POMC), transcript variant 2 | NM_000939.1 | 0.009 | 1.6 | 0.246 | 1.3 |
| similar to envelope protein (LOC113386) | NM_138781.1 | 0.043 | 3.1 | 0.246 | 1.4 |
| fibronectin type III domain containing 3A (FNDC3A), transcript variant 2 | NM_014923.2 | 0.002 | 3.1 | 0.252 | 1.2 |
| DYRK3 | PV3837 | 0.043 | 2.2 | 0.255 | 1.1 |
| **Homo sapiens, glutamate decarboxylase 2 (pancreatic islets and brain, 65kDa) (GAD2), transcript variant 1, mRNA** | **NM_000818.1** | 0.515 | 1.0 | 0.260 | 1.1 |
| ubiquitin carboxyl-terminal hydrolase L5 (UCHL5) | BC025369.1 | 0.043 | 3.3 | 0.265 | 1.5 |
| DNA helicase HEL308 (HEL308) | BC011863.2 | 0.043 | 4.1 | 0.265 | 1.2 |
| Isocitrate dehydrogenase [NADP] cytoplasmic | BC093020.1 | 0.039 | 3.2 | 0.265 | 1.2 |
| transglutaminase 2 (C polypeptide, protein-glutamine-gamma-glutamyltransferase) (TGM2) | BC003551.1 | 0.013 | 1.7 | 0.265 | 1.2 |
| chromosome 22 open reading frame 33 (C22orf33) | NM_178552.2 | 0.011 | 3.2 | 0.265 | 1.2 |
| SH3-domain GRB2-like 2 (SH3GL2) | BC032825.2 | 0.043 | 2.6 | 0.265 | 1.1 |
| guanine nucleotide binding protein (G protein), gamma 13 (GNG13) | NM_016541.1 | 0.043 | 2.8 | 0.265 | 1.2 |
| RD RNA binding protein (RDBP) | NM_002904.4 | 0.013 | 2.8 | 0.265 | 1.0 |
| Sterile alpha motif domain-containing protein 12 | NM_207506.1 | 0.010 | 6.3 | 0.311 | 1.2 |
| leukocyte receptor cluster (LRC) member 1 (LENG1) | NM_024316.1 | 0.001 | 2.6 | 0.311 | 1.0 |
| Spermatid nuclear transition protein 4 | XM_088679.2 | 0.043 | 4.7 | 0.325 | 1.0 |
| menage a trois homolog 1, cyclin H assembly factor (Xenopus laevis) (MNAT1) | NM_002431.1 | 0.011 | 2.7 | 0.325 | 1.4 |
| phospholipase A2, group IVC (cytosolic, calcium-independent) (PLA2G4C) | BC017956.1 | 0.013 | 3.3 | 0.383 | 1.1 |
| **Insulin** | **Insulin** | - | - | 0.385 | 1.1 |
| thiamin pyrophosphokinase 1 (TPK1) | BC068460.1 | 0.010 | 2.8 | 0.387 | 1.2 |
| survival of motor neuron protein interacting protein 1 (SIP1), transcript variant alpha | NM_003616.2 | 0.047 | 2.9 | 0.387 | 1.1 |
| RING finger protein 135 | NM_197939.1 | 0.047 | 2.0 | 0.407 | 1.1 |
| TGFB-induced factor homeobox 2 (TGIF2) | NM_021809.2 | 0.047 | 2.3 | 0.407 | 1.0 |
| spermidine/spermine N1-acetyltransferase 2 (SAT2) | NM_133491.2 | 0.025 | 4.3 | 0.407 | 1.3 |
| regulator of G-protein signaling 8 (RGS8), transcript variant 1 | NM_033345.1 | 0.043 | 3.1 | 0.407 | 1.0 |
| hypothetical protein MGC13057 (MGC13057), transcript variant 4 | NM_032321.1 | 0.043 | 2.7 | 0.407 | 1.3 |
| cysteine and glycine-rich protein 1 (CSRP1) | NM_004078.1 | 0.001 | 2.5 | 0.407 | 1.0 |
| family with sequence similarity 21, member C (FAM21C) | BC006456.1 | 0.011 | 4.6 | 0.413 | 1.1 |
| phosphofructokinase, liver (PFKL), transcript variant 1 | NM_001002021.1 | 0.039 | 1.6 | 0.430 | 1.0 |
| ladinin 1 (LAD1) | NM_005558.2 | 0.025 | 4.4 | 0.440 | 1.0 |
| Na+/H+ exchanger domain containing 2 (NHEDC2) | NM_178833.3 | 0.039 | 2.5 | 0.459 | 1.2 |
| cell division cycle 25 homolog A (S. pombe) (CDC25A), transcript variant 2 | NM_201567.1 | 0.009 | 2.8 | 0.459 | 1.0 |
| **Protein tyrosine phosphatase, receptor type, N** | **PTPRN (IA-2)** | - | - | 0.469 | 1.1 |
| serine/threonine kinase 33 (STK33) | BC031231.1 | 0.002 | 2.7 | 0.469 | 1.0 |
| protein kinase C, alpha (PRKCA); see catalog number for detailed information on wild-type or point mutant status | NM_002737.1 | 0.047 | 2.9 | 0.474 | 0.8 |
| **Solute carrier family 30 (zinc transporter), member 8** | **SLC30A8 (ZnT8)** | - | - | 0.493 | 0.9 |
| ribosomal protein S6 kinase, 70kDa, polypeptide 1 (RPS6KB1) | BC053365.1 | 0.043 | 2.1 | 0.493 | 1.2 |
| Phospholipase DDHD2 | NM_015214.1 | 0.043 | 2.7 | 0.494 | 1.1 |
| excision repair cross-complementing rodent repair deficiency, complementation group 1 (includes overlapping antisense sequence) (ERCC1), transcript variant 2 | NM_001983.1 | 0.039 | 2.6 | 0.494 | 1.0 |
| NEK1 | PV4202 | 0.002 | 2.7 | 0.494 | 1.0 |
| aryl-hydrocarbon receptor nuclear translocator 2 (ARNT2) | BC036099.1 | 0.047 | 2.5 | 0.494 | 1.1 |
| RAB37, member RAS oncogene family (RAB37), transcript variant 3 | NM_175738.2 | 0.043 | 3.6 | 0.494 | 1.0 |
| T-box transcription factor TBX20 | NM_020417.1 | 0.011 | 2.8 | 0.494 | 1.0 |
| Autophagy-related protein 16-1 | NM_017974.2 | 0.043 | 2.6 | 0.494 | 1.0 |
| Phosphoglycerate mutase 2 | BC001904.1 | 0.010 | 3.9 | 0.494 | 1.0 |
| sterol carrier protein 2 (SCP2) | BC005911.1 | 0.043 | 1.8 | 0.494 | 1.0 |
| UNC-112 related protein 2 (URP2) | BC013366.2 | 0.013 | 2.9 | 0.494 | 1.0 |
| ABL1 proto-oncogene | PV3866 | 0.047 | 2.5 | 0.494 | 1.1 |
